# Supplementary material for: GSTM5 as a Potential Biomarker for Treatment Resistance in Prostate Cancer
Source: Biomedicines. 2025 Aug 1;13(8):1872. doi: 10.3390/biomedicines13081872 (PMC12383628; doi:10.3390/biomedicines13081872)
Supplement: Supplementary file 1 [file biomedicines-13-01872-s001.zip › biomedicines-3597923-supplementary.pdf]

GSTM5 as a potential biomarker for treatment resistance in prostate cancer

Authors: Porras-Quesada Patricia [1,2,†], Chica-Redecillas Lucía [1,2,†], Álvarez-González Beatriz [2,3,*], Gutiérrez-Tejero Francisco[4], Arrabal-Martín Miguel[4,5], Rios-Pelegrina Rosa[5,6], Martínez-González Luis Javier[1,2,5†], Álvarez-Cubero María Jesús[1,2,5†], Vázquez-Alonso Fernando[7]

Affiliations:

[1] University of Granada, Biochemistry and Molecular Biology III and Immunology Department, Faculty of Medicine, PTS, Granada, Spain
[2] GENYO, Centre for Genomics and Oncological Research: Pfizer, University of Granada, Andalusian Regional Government, PTS Granada, Granada, Spain
[3] University of Granada, Legal Medicine and Toxicology Department, Faculty of Medicine, PTS, Granada, Spain
[4] San Cecilio University Hospital, Department of Urology, Granada, Spain
[5] Biosanitary Research Institute (ibs. GRANADA), University of Granada, Granada, Spain
[6] University Hospital Virgen de las Nieves, Department of Pathology, Granada, Spain
[7] University Hospital Virgen de las Nieves, Urology Department, Granada, Spain
* Correspondence: bealvarez@ugr.es. +34 958 248 945. B. Álvarez-González. PTS Granada; Av. Doctor Jesús Candel Fábregas, 11, 18016 Granada, Spain
† These authors contributed equally to this work

Contact information corresponding author: mjesusac@ugr.es. +34 958 248 945. MJ Álvarez-Cubero. PTS Granada; Av. Doctor Jesús Candel Fábregas, 11, 18016 Granada, Spain.

Keywords: Castration-Resistant Prostate Cancer, *GSTM5*, Biomarker

Table S1. KEGG Pathway Enrichment Analysis of *GSTM5* and its 15 Top Interactors

Kegg Pathway	p-value	FDR	Gene count	Genes involved
Chemical carcinogenesis - DNA adducts	2.58E-20	7.74E-19	11	*CYP1A2, CYP3A4, GSTM4, CYP2E1, GSTM3, EPHX1, GSTM2, **GSTM5**, CYP1B1, CYP1A1, CYP2A13*
Metabolism of xenobiotics by cytochrome P450	7.93E-20	1.19E-18	11	*CYP1A2, CYP3A4, GSTM4, CYP2E1, GSTM3, EPHX1, GSTM2, **GSTM5**, CYP1B1, CYP1A1, CYP2A13*
Glutathione metabolism	3.94E-14	3.94E-13	8	*GSR, GSTM4, GSTM3, GSTM2, **GSTM5**, PRDX6, GPX4, GSS*
Chemical carcinogenesis - receptor activation	3.38E-11	1.76E-10	9	*CYP1A2, CYP3A4, GSTM4, GSTM3, EPHX1, GSTM2, **GSTM5**, CYP1B1, CYP1A1*
Drug metabolism - cytochrome P450	2.50E-11	1.76E-10	7	*CYP1A2, CYP3A4, GSTM4, CYP2E1, GSTM3, GSTM2, **GSTM5***
Chemical carcinogenesis - reactive oxygen species	3.53E-11	1.76E-10	9	*CYP1A2, GSTM4, CYP2E1, GSTM3, EPHX1, GSTM2, **GSTM5**, CYP1B1, CYP1A1*
Drug metabolism - other enzymes	5.02E-09	2.15E-08	6	*CYP3A4, GSTM4, CYP2E1, GSTM3, GSTM2, **GSTM5***
Steroid hormone biosynthesis	7.73E-08	2.79E-07	5	*CYP1A2, CYP3A4, CYP2E1, CYP1B1, CYP1A1*
Metabolic pathways	8.37E-08	2.79E-07	13	*GSR, CYP1A2, CYP3A4, GSTM4, CYP2E1, GSTM3, GSTM2, **GSTM5**, GSTZ1, PRDX6, CYP1A1, GPX4, GSS*
Platinum drug resistance	9.94E-06	2.98E-05	4	*GSTM4, GSTM3, GSTM2, **GSTM5***
Linoleic acid metabolism	2.25E-05	6.14E-05	3	*CYP1A2, CYP3A4, CYP2E1*
Tryptophan metabolism	6.27E-05	0.00015681	3	*CYP1A2, CYP1B1, CYP1A1*
Fluid shear stress and atherosclerosis	0.00011961	0.000276029	4	*GSTM4, GSTM3, GSTM2, **GSTM5***
Hepatocellular carcinoma	0.00024623	0.000527632	4	*GSTM4, GSTM3, GSTM2, **GSTM5***
Retinol metabolism	0.00026564	0.000531289	3	*CYP1A2, CYP3A4, CYP1A1*
Ferroptosis	0.00281668	0.005281282	2	*GPX4, GSS*
Ovarian steroidogenesis	0.00412949	0.007287339	2	*CYP1B1, CYP1A1*
Arachidonic acid metabolism	0.0058615	0.009769158	2	*CYP2E1, GPX4*
Caffeine metabolism	0.01140876	0.018013827	1	*CYP1A2*
Bile secretion	0.01242139	0.01863209	2	*CYP3A4, EPHX1*
Pathways in cancer	0.01596268	0.022803833	4	*GSTM4, GSTM3, GSTM2, **GSTM5***

Foot Table S1. FDR = False Discovery Rate

Table S2. Association of *GSTM5* rs3768490 Genotype and Clinical Variables: *Long-term responders* vs *Early-onset CRPC*

	Early-onset *CRPC* N = 54 (%)	*Long-term responders* N = 67 (%)	Contingency test*	Simple logistic regression	
			p-value	OR (95% CI)	*p*-value
Genotype			0.0721		
TT	2 (1.6%)	10 (8.3%)		0.18 (0.02 - 0.76)	**0.0359**
GT	27 (22.3%)	35 (28.9%)		0.68 (0.31 - 1.45)	0.3190
GG	25 (20.7%)	22 (18.2%)		Ref.	
Dominant model			0.1860		
GT+TT	29 (24.0%)	45 (37.2%)		0.57 (0.27 - 1.18)	0.1325
GG	25 (20.7%)	22 (18.2%)		Ref.	
Recessive model			0.0636		
GG+GT	52 (43.0%)	57 (47.1%)		Ref.	
TT	2 (1.6%)	10 (8.3%)		0.22 (0.03 - 0.88)	0.0572
Overdominant model			0.9506		
GG+TT	27 (22.3%)	32 (26.4%)		Ref.	
GT	27 (22.3%)	35 (29.0%)		0.91 (0.44 - 1.87)	0.8065
PSA Level			0.3050		
<10 ng/mL	15 (12.8%)	25 (21.4%)		Ref.	
>10 ng/mL	38 (32.5%)	39 (33.3%)		1.62 (0.75 - 3.56)	0.2235
Gleason Score			**0.0001**		
<7	12 (10.2%)	41 (35.0%)		Ref.	
≥7	39 (33.3%)	25 (21.4%)		5.33 (2.41 – 12.43)	**0.0001**
T Stage (TNM)			**0.0005**		
T1-T2	23 (24.7%)	48 (51.6%)		Ref.	
T3-T4	17 (18.3%)	5 (5.4%)		7.09 (2.47 - 23.81)	**0.0006**
D'Amico risk			**0.0004**		
Low-Mid	6 (5.0%)	27 (22.7%)		Ref.	
High	47 (39.5%)	39 (32.8%)		5.42 (2.15 - 15.74)	**0.0007**

Table S3. Association of *GSTM5* rs3768490 Genotype and Clinical Variables: *Non-progression responders* vs *Early-onset CRPC.*

	Early-onset CRPC N = 54 (%)	*Non-progression responders* N = 64 (%)	Contingency test*	Simple logistic regression	
			p-value	OR (95% CI)	*p*-value
Genotype			0.0795		
TT	2 (1.7%)	10 (8.5%)		0.18 (0.02 - 0.76)	**0.0359**
GT	27 (22.9%)	32 (27.1%)		0.74 (0.34 - 1.60)	0.4477
GG	25 (21.2%)	22 (18.6%)		Ref.	
Dominant model			0.2588		
GT+TT	29 (24.6%)	42 (35.6%)		0.61 (0.29 - 1.27)	0.1888
GG	25 (21.2%)	22 (18.4)		Ref.	
Recessive model			**0.0369**		
GG+GT	52 (44.1%)	54 (45.8%)		Ref.	
TT	2 (1.7%)	10 (8.5%)		0.21 (0.03 - 0.83)	**0.0491**
Overdominant model			1.0000		
GG+TT	27 (22.9%)	32 (27.1%)		Ref.	
GT	27 (22.9%)	32 (27.1%)		1 (0.48 - 2.07)	1.0000
PSA Level			0.3881		
<10 ng/dL	15 (13.2%)	23 (20.2%)		Ref.	
>10 ng/dL	38 (33.3%)	38 (33.3%)		1.53 (0.70 - 3.43)	0.2894
Gleason Score			**4.7E-05**		
<7	12 (10.5%)	40 (35.1%)		Ref.	
≥7	39 (34.2%)	23 (20.2%)		5.65 (2.53 - 13-32)	**3.9E-05**
T Stage (TNM)			**0.0006**		
T1-T2	23 (25.0%)	47 (51.1%)		Ref.	
T3-T4	17 (18.5%)	5 (5.4%)		6.95 (2.42 - 23.33)	**0.0007**
D'Amico risk			**0.0003**		
Low-Mid	6 (5.2%)	26 (22.4%)		Ref.	
High	47 (40.2%)	37 (31.9%)		5.50 (2.17 - 16.05)	**0.0007**

Table S4. Multiple logistic regression analysis for ADT response in PC.

Comparison	Variable	*p*-value	*OR (95% IC)*
**Early-onset CRPC vs. Long-Term responders**	TT Genotype (Ref. GG)	**0.0487**	0.18 (0.02 - 0.86)
	GT Genotype (Ref. GG)	0.5451	0.77 (0.33 - 1.78)
	High D'Amico risk (Ref. Low-Mid)	**0.0005**	5.83 (2.27 - 17.17)
	Recessive model (TT vs. GG+GT)	0.0604	0.21 (0.03 - 0.92)
	High D'Amico risk (Ref. Low-Mid)	**0.0005**	5.90 (2.30 - 17.36)
**Early-onset CRPC vs. Non-progression responders**	TT Genotype (Ref. GG)	**0.0484**	0.18 (0.02 - 0.86)
	GT Genotype (Ref. GG)	0.7238	0.86 (0.37 - 1.99)
	High D'Amico risk (Ref. Low-Mid)	**0.0005**	5.99 (2.31 - 17.76)
	Recessive model (TT vs. GG+GT)	0.0509	0.19 (0.03 - 0.86)
	High D'Amico risk (Ref. Low-Mid)	**0.0004**	6.04 (2.34 - 17.90)

Study group (n)	*GSTM5* expression (Median (95% CI))	p-value[a]
GSTM5 genotype:		
G/G (52)	0.7377 (0.4078 - 0.9254)	0.0635
G/T (43)	0.4967 (0.2260 - 0.8691)	
T/T (17)	0.9736 (0.5797 - 1.3760)	
GSTM5 genotype:		
G/G – G/T (95)	0.5764 (0.3803 - 0.8511)	0.0629
T/T (17)	0.9736 (0.5797 - 1.3760)	
ADT response:		
Early-onset CRPC (5)	0.1193 (0.0315 – 0.4319)	0.0790
Long-term responders (16)	0.5451 (0.0739 – 1.2280)	
ADT response:		
Early-onset CRPC (5)	0.1193 (0.0315 – 0.4319)	0.3420
Non-progression responders (21)	0.6782 (0.0955 – 1.2510)	
D'Amico Risk:		
Low - Intermediate (78)	0.7961 (0.5274 – 1.1880)	0.1253
High (34)	0.6217 (0.1851 – 0.9555)	

Table S5. Statistical report of *GSTM5* expression analysis

Foot Foot Table S5. [a] p-value obtained by the Kruskal-Wallis test for comparisons between three groups and the Mann-Whitney test for pairwise comparisons between two groups.
